# Supplementary material for: End Sequence Analysis Toolkit (ESAT) expands the extractable information from single-cell RNA-seq data
Source: Genome Res. 2016 Oct;26(10):1397–410. doi: 10.1101/gr.207902.116 (PMC5052061; doi:10.1101/gr.207902.116)
Supplement: Supplemental Material [file supp_26_10_1397__index.html]

End Sequence Analysis Toolkit (ESAT) expands the extractable information from single-cell RNA-seq data — Supplemental Material 

# End Sequence Analysis Toolkit (ESAT) expands the extractable information from single-cell RNA-seq data

## Supplemental Material

- Supplemental\_Fig\_S1.pdf
- Supplemental\_Fig\_S2.eps
- Supplemental\_Fig\_S3.pdf
- Supplemental\_Fig\_S4.eps
- Supplemental\_Fig\_S5.pdf
- Supplemental\_Fig\_S6.eps
- Supplemental\_Fig\_S7.pdf
- Supplemental\_Table\_S1.xlsx
- Supplemental\_Table\_S2.xlsx
- Supplemental\_Table\_S3.xlsx
- Supp\_Materials.tar.gz
- Supplemental\_data\_S1.tar.gz
