# Supplementary material for: End Sequence Analysis Toolkit (ESAT) expands the extractable information from single-cell RNA-seq data
Source: Genome Res. 2016 Oct;26(10):1397–410. doi: 10.1101/gr.207902.116 (PMC5052061; doi:10.1101/gr.207902.116)
Supplement: Supplemental Material [file supp_gr.207902.116_Supplemental_Fig_S1.pdf]

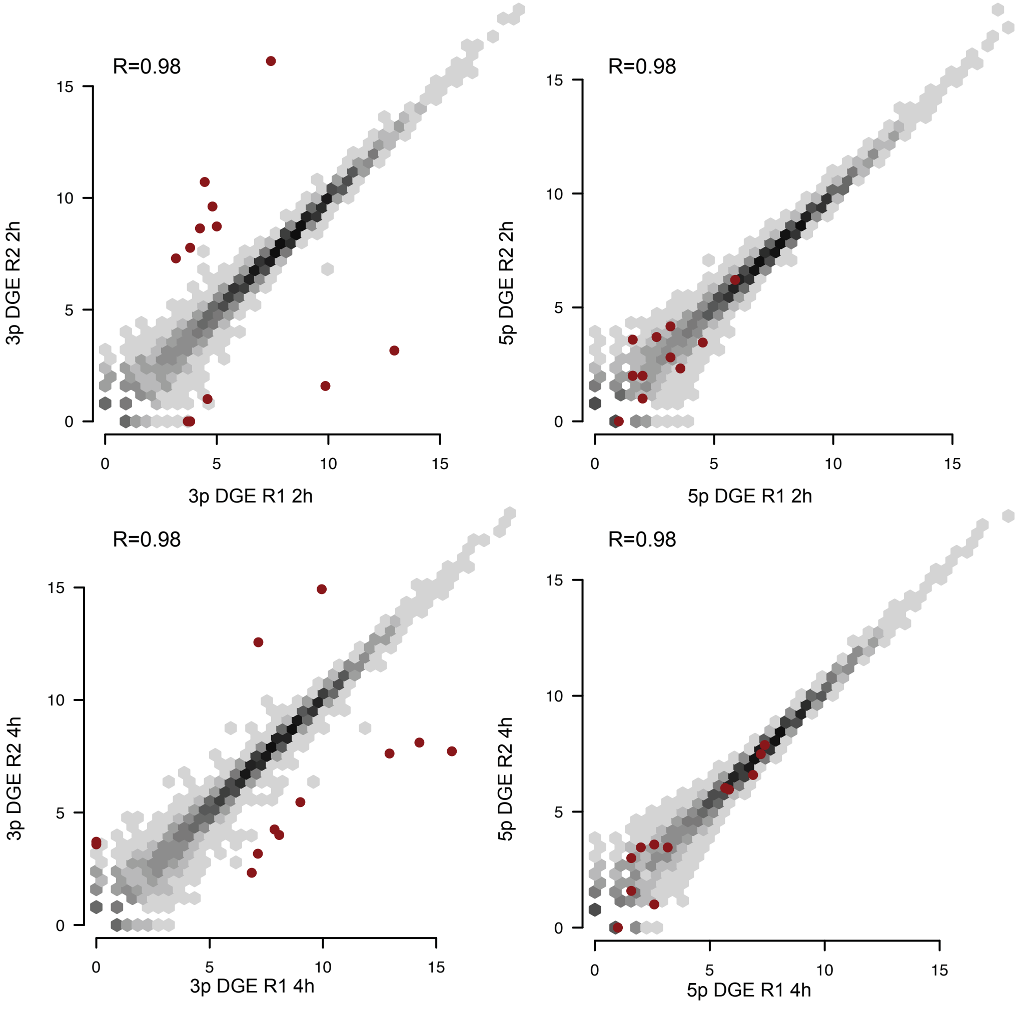

Supplemental Fig S1 - End-sequencing is highly reproducible. Technical replicates of 5' and 3' libraries of LPS stimulated DCs after 2 and 4 hours. Red dots highlight outliers (at least 10 fold difference between replicates in 3' libraries)
