# Supplementary material for: End Sequence Analysis Toolkit (ESAT) expands the extractable information from single-cell RNA-seq data
Source: Genome Res. 2016 Oct;26(10):1397–410. doi: 10.1101/gr.207902.116 (PMC5052061; doi:10.1101/gr.207902.116)
Supplement: Supplemental Material [file supp_gr.207902.116_Supplemental_Fig_S5.pdf]

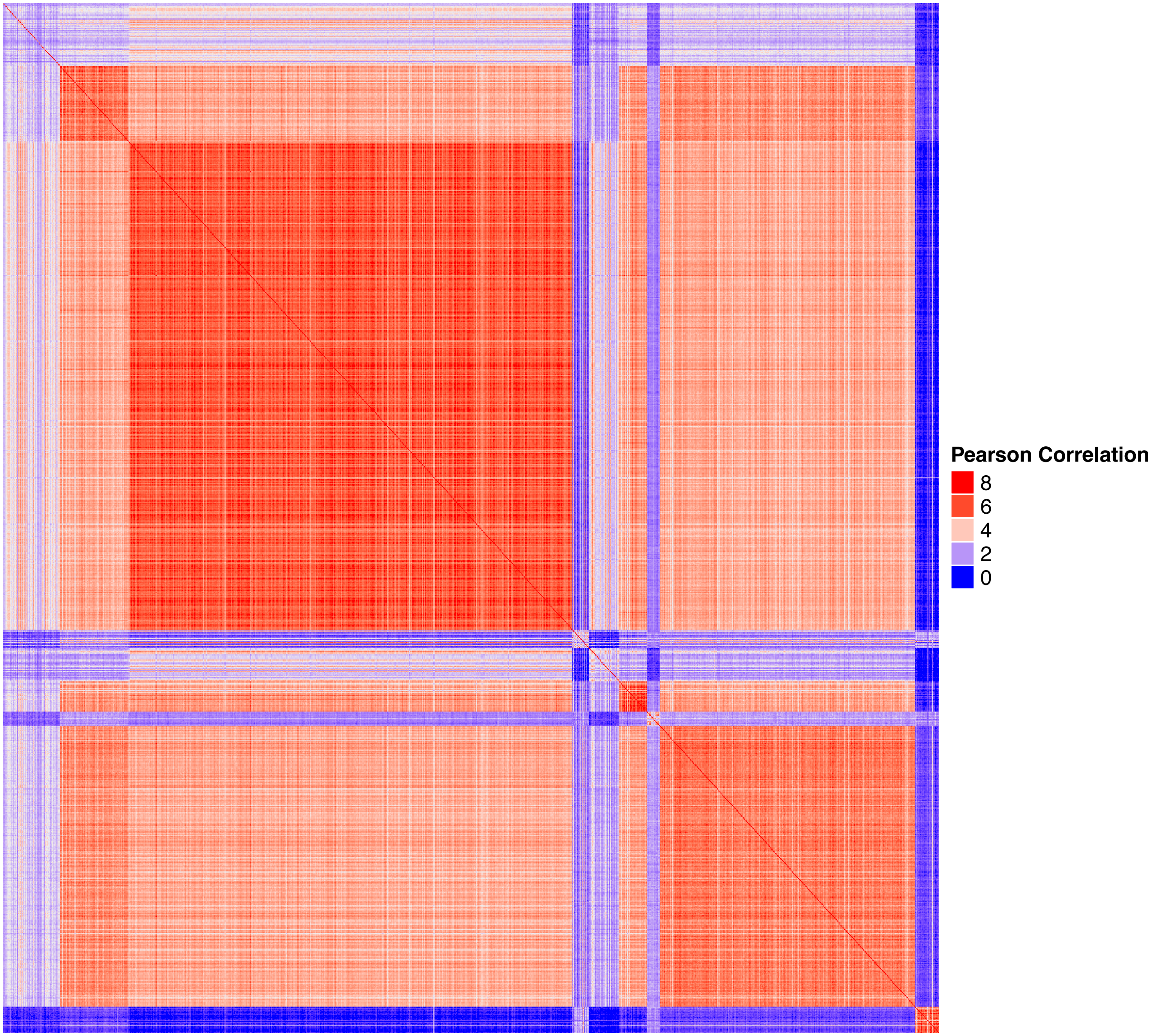

Supplemental Fig S4. No global switch to shorter 5' TSS expression in stimulated DCs. Boxplots of the fraction of transcripts expressing the earliest TSS for genes with detectable expression of at least two distinct 5' TSSs in unstimulated DCs (total of 26). p-values were computed using a Mann-Whitney rank sum test between the two unstimulated distributions and each of the time points shown.
