# Supplementary material for: End Sequence Analysis Toolkit (ESAT) expands the extractable information from single-cell RNA-seq data
Source: Genome Res. 2016 Oct;26(10):1397–410. doi: 10.1101/gr.207902.116 (PMC5052061; doi:10.1101/gr.207902.116)
Supplement: Supplemental Material [file supp_gr.207902.116_Supplemental_Fig_S7.pdf]

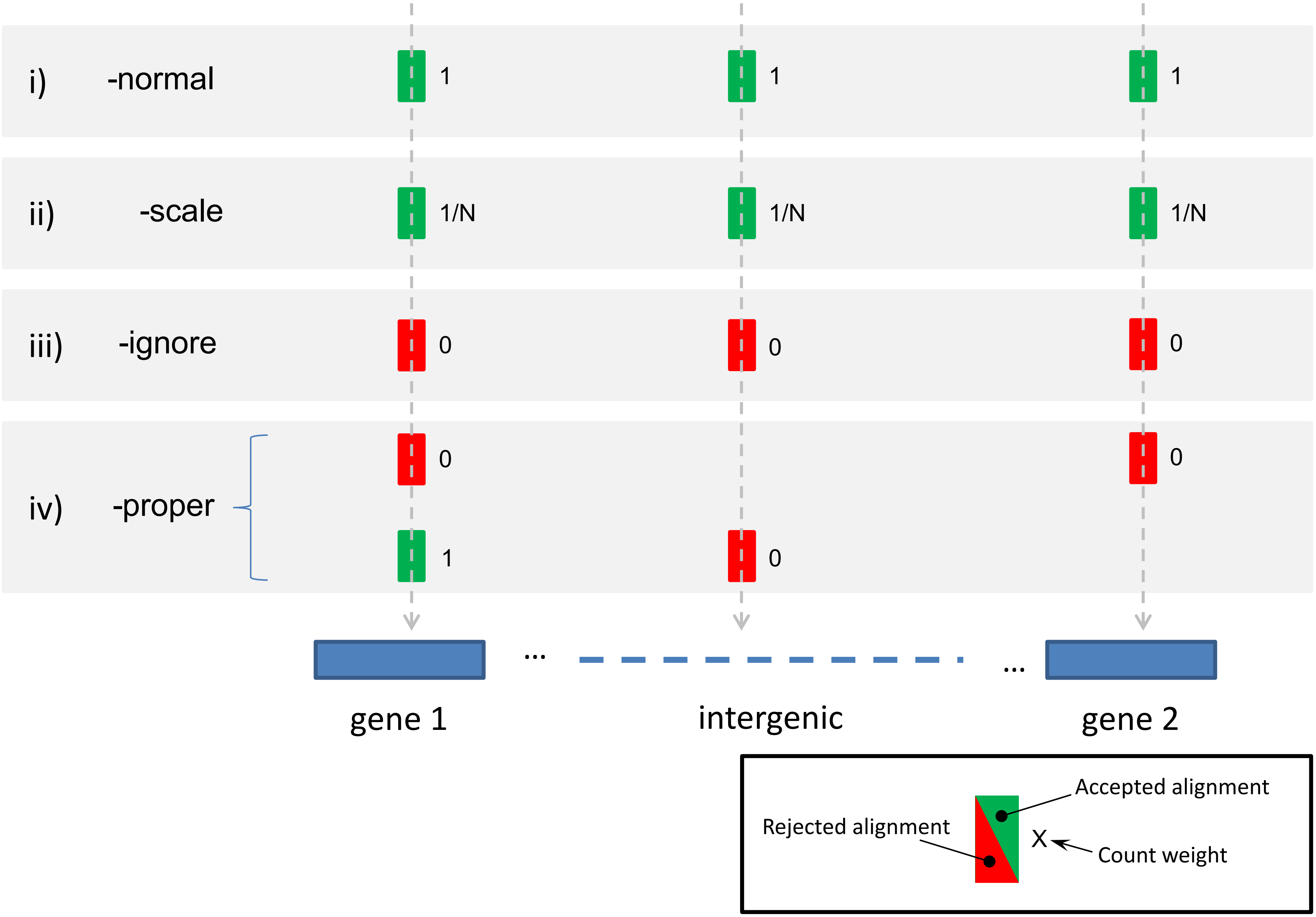

Supplemental Fig S7. Multimapper handling method. A) -normal counts each multimapped read with a full count. B) -scale weights each of N multimapped reads by 1/N. C) -ignore discards all multmapped reads. D) -proper multimapped reads are processed according to the algorithm described in the text.
