# Supplementary material for: End Sequence Analysis Toolkit (ESAT) expands the extractable information from single-cell RNA-seq data
Source: Genome Res. 2016 Oct;26(10):1397–410. doi: 10.1101/gr.207902.116 (PMC5052061; doi:10.1101/gr.207902.116)
Supplement: Supplemental Material [file supp_gr.207902.116_Supp_Materials.tar.gz › ESAT-esat_v0.1/doc/html/_command_line_parser_8java.html]

ESAT: java/broad/core/parser/CommandLineParser.java File Reference


|  |
| --- |
| ESAT |


- Main Page
- Related Pages
- Packages
- Classes
- Files

- File List

All Classes Namespaces Files Functions Variables Enumerator Pages

- java
- broad
- core
- parser

Classes |
Packages

CommandLineParser.java File Reference

|  |  |
| --- | --- |
| Classes | |
| class | broad.core.parser.CommandLineParser |
|  | |

|  |  |
| --- | --- |
| Packages | |
| package | broad.core.parser |
|  | |


---

Generated on Fri Aug 15 2014 15:10:48 for ESAT by  

 1.8.7
