# Supplementary material for: End Sequence Analysis Toolkit (ESAT) expands the extractable information from single-cell RNA-seq data
Source: Genome Res. 2016 Oct;26(10):1397–410. doi: 10.1101/gr.207902.116 (PMC5052061; doi:10.1101/gr.207902.116)
Supplement: Supplemental Material [file supp_gr.207902.116_Supplemental_Fig_S3.pdf]

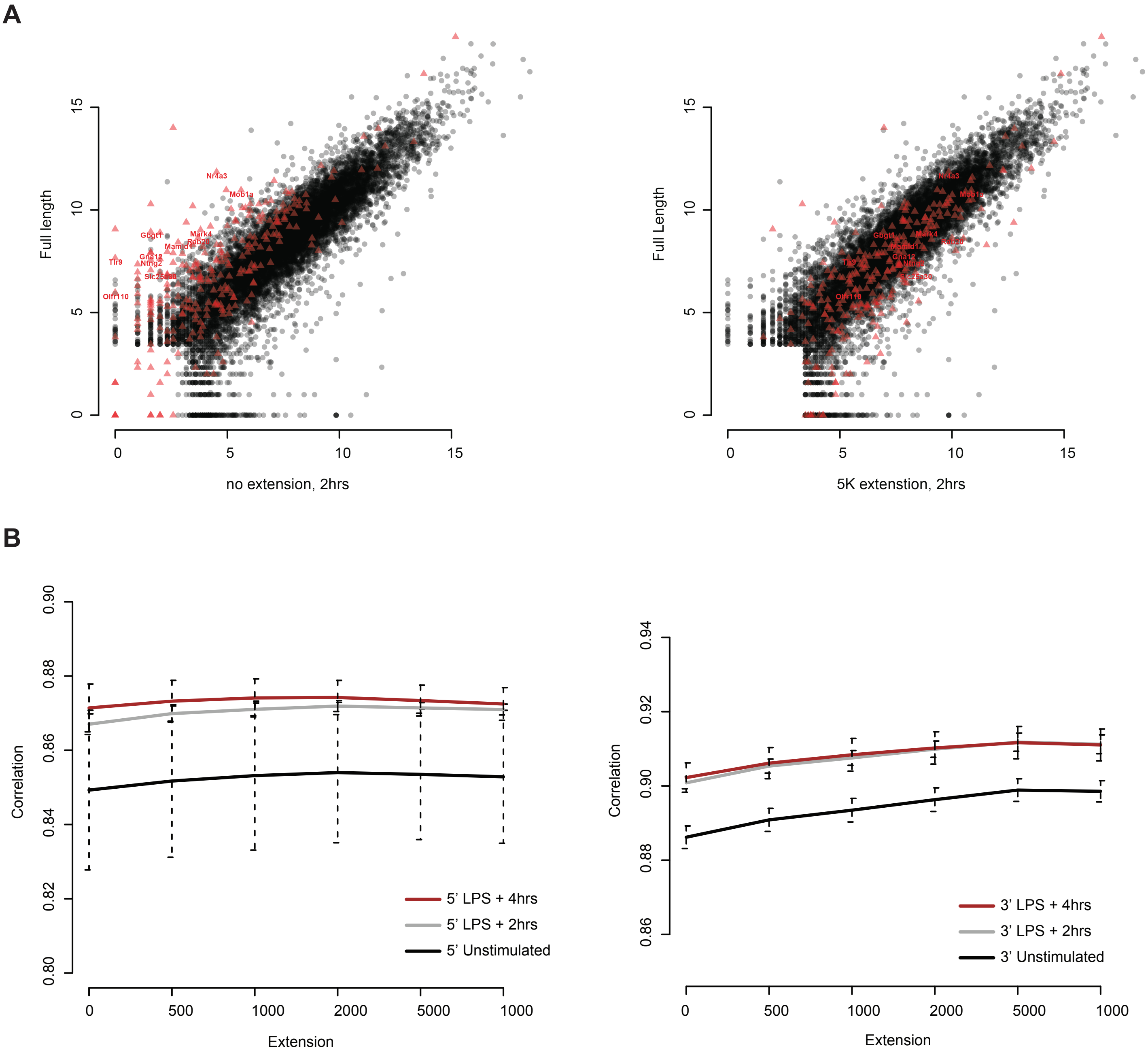

Supplemental Fig S3. Extension and multimapper handling increases power without adding noise. A) Comparison of the LPS + 2 hour library (coding genes only) with the full length before and after a 2 kb extension. Red points indicate genes for which the fold change difference between the full and 3' library was greater than 4.B) For each library we the plot shows the correlation of estimated gene expression for 3' (left panel) and 5' (right panel) libraries after extending genes by the indicated amount on the xaxis with the full length library from garber et al (Garber et al. 2012).
